# The Effect of Melaxen on the Activity of Caspases and the Glutathione Antioxidant System in Toxic Liver Injury

**Published:** 2014

**Authors:** S. S. Popov, K. K. Shulgin, A. N. Pashkov, A. A. Agarkov

**Affiliations:** N.N. Burdenko Voronezh State Medical Academy, Ministry of Health of the Russian Federation, Studencheskaya Str. 10, Voronezh, Russia, 394036; Voronezh State University, Ministry of Education and Science of the Russian Federation, Universitetskaya Str. 1, Voronezh, Russia, 394006

**Keywords:** chronic alcoholic hepatitis, glutathione peroxidase, glutathione reductase, reduced glutathione, glutathione-S-transferase, caspases, melaxen

## Abstract

A comparative study of the activity of caspase-1 and caspase-3, the glutathione antioxidant system and NADPH-generating enzymes (glucose-6-phosphate dehydrogenase and NADP-isocitrate dehydrogenase) and a study of DNA fragmentation in the blood serum of patients with chronic alcoholic hepatitis during basic treatment and combination therapy including melaxen have been carried out. It was found that the blood serum level of reduced glutathione, which decreases in pathology, increased more significantly in patients receiving melaxen as compared to the group of patients receiving the standard treatment. More significant changes in the activity of caspase-1 and caspase-3, glutathione reductase, glutathione peroxidase, glutathione-S-transferase, glucose-6-phosphate dehydrogenase and NADP-isocitrate dehydrogenase toward the control values were observed during the combination therapy. The correction in the melatonin level under the influence of melaxen apparently had a positive effect on the free-radical homeostasis in patients, which resulted in more pronounced changes in the investigated parameters towards the normal values as compared to the basic treatment. KEY WORD S chronic alcoholic hepatitis; glutathione peroxidase; glutathione reductase; reduced glutathione; glutathione-S-transferase; caspases; melaxen.

## INTRODUCTION

Toxic liver injury usually develops after 5–10 years of alcohol abuse and is characterized by necrosis, along with an inflammatory reaction. The characteristic features of liver damage in patients include the prevalence of steatosis and other abnormalities in the perivascular (centrilobular) acinar zone. The mechanism of this zonal selectivity is associated with a relative oxygen deficiency. Low oxygen tension increases the redox potential shift caused by ethanol. Ethanol increases the lactate/ pyruvate ratio and decreases the pyruvate level more significantly in the venous blood of the liver than in the entire body. Hypoxia leads to an increase in the NADH level, dysfunction of some enzymes, formation of oxygen radicals, and activation of lipid peroxidation (LPO) [1]. It is known that alcohol-induced cytochrome-P450- monooxygenase (CYPE1) catalyzes the oxidation of ethanol, which contributes to the growth of tolerance to alcohol, as well as its transformation into highly toxic metabolites, including reactive oxygen species (ROS). Depletion of the reduced glutathione (GSH) level, which occurs under these circumstances, induces oxidative stress and damage to liver cells. Disturbance of the redox homeostasis in toxic liver injury can cause the activation of programmed cell death (apoptosis), which is characterized by the activation of the cascade of intracellular cysteine proteases known as caspases [2]. It is believed that activation of caspases is a key step in the intermediate and terminal stages of this process [3]. Thus, caspase-3, which belongs to the ced-3 family, is directly involved in apoptosis and is capable of activating other caspases. Then the process of programmed cell death becomes irreversible. We cannot exclude the participation of caspase-1, which belongs to the ICE family and is involved in the processing of cytokines [3], in apoptotic cell death. For example, caspase-1 expression was observed in the atrophic acinar cells of the pancreas in patients with chronic pancreatitis. This fact is indicative of their death. Moreover, caspase-1 promotes caspase-3 activation [3].

It is known that GSH and the enzymes associated with its transformations play an important role in protecting the body against both ROS and toxic substances. GSH belongs to the most important group of toxicity control agents. It is capable of reacting with free radicals, in particular, neutralizing singlet oxygen and hydroxyl radicals, and inhibiting LPO processes [4]. The glutathione reductase/glutathione peroxidase (GR [EC 1.6.4.2.]; GP [EC 1.11.1.9.]) system performs the detoxification of H_2_O_2_ and hydroperoxides using GSH, due to the action of glutathione peroxidase. The rate of GSH formation in the coupled reaction catalyzed by glutathione reductase mainly depends on the NADPH level [5]. The pentose phosphate pathway with glucose- 6-phosphate dehydrogenase (G6PDG [EC 1.1.1.49.]) being its key enzyme catalyzing the conversion of glucose- 6-phosphate to 6-phosphogluconolactone is one of the major suppliers of NADPH to the GR/GP-system [6]. The reaction catalyzed by NADP-isocitrate dehydrogenase (NADP-IDG [EC 1.1.1.42.]), which includes the oxidative decarboxylation of isocitrate to 2-oxoglutarate [7], can be an alternative source of NADPH. The glutathione antioxidant system (AOS) also includes glutathione-S-transferases (GST), the multifunctional proteins that use GSH for the metabolism of many hydrophobic substances and perform the detoxification of xenobiotics [8]. GST protects DNA, mitochondria, and other vital cell components against toxic substances and, thus, significantly increases the resistance of cells and the organism as a whole [9].

Melatonin, a hormone of the diffuse neuroendocrine system, which regulates several physiological functions, belongs to antioxidants. Melatonin is involved in the formation of circadian rhythms, suppression of some pituitary functions, and regulation of immune responses. According to its chemical structure, melatonin (N-acetyl-5-methoxytryptamine) is a derivative of serotonin, the biogenic amine which is in turn synthesized from tryptophan amino acid [10, 11]. There is evidence that melatonin can act as an interceptor of the hydroxyl radical, singlet oxygen, and nitric oxide [12]. Furthermore, melatonin facilitates the expression of the genes that are responsible for the synthesis of Cu- Zn-dependent superoxide dismutase [13]. It is believed that melatonin mainly protects DNA against free radicals, although it has a significant protective effect on other macromolecules. Owing to its lipophilic properties, melatonin can easily penetrate into all organs and tissues, where its antioxidant activity can be implemented [14]. We have previously found that exogenous melatonin inhibits the development of oxidative stress in rats with toxic hepatitis [15], type 2 diabetes mellitus [16], and hyperthyroidism [17]. In this study, we have used melaxen, a synthetic drug containing melatonin, in the treatment of patients with toxic liver damage caused by excessive alcohol consumption.

This study was aimed at a comparative evaluation of the activity of caspase-1, caspase-3, GR, GP, GST, NADPH-generating enzymes (G6PDG and NADPIDG), the GSH content, and the degree of DNA fragmentation in the blood of patients in the acute stage of chronic alcoholic hepatitis (CAH) during basic treatment and combination therapy including melaxen.

## EXPERIMENTAL

The clinical study included 52 patients with toxic liver injury caused by chronic alcohol abuse. All patients were males aged 22–69 years, mean age 41.4 ± 7.2 years. All of them suffered from the alcohol dependence syndrome. The average duration of the disease was 2.2 ± 0.5 months. Alcoholic hepatitis was diagnosed based on clinical symptoms, biochemical blood tests, and hepatic ultrasound findings. The most common comorbidities included chronic gastritis – 32 patients (50%) and hypertension – 24 patients (30.5%).

The control group included 65 apparently healthy subjects with normal clinical and biochemical blood tests.

Viral hepatitis, cancer, diabetes mellitus, acute myocardial infarction, and cerebrovascular accident were the exclusion criteria.

The patients were divided into two groups. The first group (28 patients) received a basic treatment including complete alcohol withdrawal, diet number 5, 0.9% NaCl solution and vitamin B1 solution (10 ml) intravenously, riboxinum solution (10 ml) intravenously, vitamin B6 solution (4 ml) intramuscularly, and relanium solution (4 ml) intravenously. Hepatoprotectors: carsil (equivalent to 35 mg of silymarin) two tablets three times a day at mealtimes, Essliver Forte (essential phospholipids 300 mg) two tablets three times a day for 10 days. The second group (24 patients) in addition to the basic treatment received melaxen (Unifarm, Inc., USA) one tablet containing 3 mg of melatonin once a day 30–40 minutes before bedtime for 10 days.

The activities of caspase-1 and caspase-3 were determined using the Caspase 1 Assay Kit, Colorimetric and Caspase 3 Assay Kit, and Colorimetric (Sigma). A cocktail of protease inhibitors (0.08 mM aprotinin, 1.5 mM pepstatin A, and 2 mM leupeptin) was added to the measurement environment at a ratio of 100:1 (all reagents produced by Sigma, USA). The colorimetric analysis of caspase activity is based on hydrolysis of the Acetyl-Tyr-Val-Ala-Asp-p-nitroanilide (Ac-YVAD-pNA) peptide substrate (for caspase- 1) and acetyl-Asp-Glu-Val-Asp-p-nitroanilide (Ac-DEVD-pNA) peptide substrate (for caspase-3) to form a p-nitroanilide residue that has an absorption maximum at 405 nm (the molar absorption factor = 10.5 M^-1^ cm^-1^). Caspase activity was expressed in pmoles of the product formed during 1 min per 1 mg of protein.

DNA was isolated from blood leukocytes using the phenol-chloroform method [18]. DNA fragmentation was detected by electrophoresis in an agarose gel in a TAE (Tris-acetate-EDTA) buffer containing ethidium bromide [19]. A MassRuler kit comprising markers from 1,500 to 10,000 bp (Fermentas, Lithuania) was used as a molecular weight marker.

The activities of glutathione AOS enzymes and NADPH-generating enzymes were determined spectrophotometrically at 340 nm on a Hitachi U-1900 spectrophotometer (Japan). The amount of the enzyme catalyzing the transformation of 1 micromole of the substrate during 1 min at 25°C was taken as the activity unit. The activity was calculated per 1 ml of blood serum. GR activity was determined in a medium containing a 50 mM potassium phosphate buffer (pH 7.4), 1 mM EDTA, 0.16 mM NADPH, and 0.8 mM oxidized glutathione. GP activity was determined in a medium containing a 50 mM potassium phosphate buffer (pH 7.4), 1 mM EDTA, 0.12 mM NADPH, 0.85 mM GSH, 0.37 mM H_2_O_2_, and 1 unit/ml GR. GST activity was determined using the method based on the assessment of the rate of glutathione-S-2,4-dinitrobenzene formation in the reaction of GSH with 1-chloro-2,4-dinitrobenzene. GST activity was measured in the following medium: a 0.1 M potassium phosphate buffer (pH 7.4), 1 mM EDTA, 1 mM 1-chloro-2,4-dinitrobenzene, and 5 mM GSH. G6PDG activity was determined spectrophotometrically in the following medium (mM): a 0.05 Tris- HCl buffer (pH 7.8), 3.2 glucose-6-phosphate, and 0.25 NADP. NADP-IDG activity was determined in a 50 mM Tris-HCl buffer (pH 7.8) containing 1.5 mM isocitrate, 0.25 mM NADP, and 1.5 mM MnCl_2_. GSH concentration was determined using a reaction with 5,5-dithiobis(2- nitrobenzoic) acid, which results in the formation of thionitrophenyl anion (TN PA) with the absorption maximum at 412 nm [ 20]. Total protein was determined by a standardized biuret test [21]. The activity of γ-glutamyl transpeptidase (γ-GTP) was evaluated according to the rate of the glutamyl residue transfer reaction from γ-L-(+)-glutamyl-4-nitroanilide to glycylglycine (Biotest, PLIVA – Lachema Diagnostika). The activities of the marker enzymes of hepatocyte damage (ALT, AST) were determined along with the standard parameters of the biochemical blood test on a Klima 15MC biochemical analyzer (Spain).

The Caspase 1 Assay Kit, Colorimetric and Caspase 3 Assay Kit, Colorimetric, isocitrate, glutathione reductase preparation, Tris-Acetate-EDTA, ethidium bromide (Sigma, USA), NADP, NADPN, Tris-HCl buffer, EDTA (Reanal, Hungary), oxidized and reduced glutathione, and glucose-6-phosphate (ICN , USA) were used in this study. The rest of the reagents used were reagent grade or analytical-reagent grade chemicals produced in the Russian Federation.

Statistical processing of the material included the standard analysis of variance methods (calculation of mean values (M), error of the mean values (m), Student’s t-test) and the non-parametric Wilcoxon test using the STATISTICA 6.0 software. The differences were considered to be statistically significant at p ≤ 0.05.

## RESULTS

γ-GTP activity was on average 3.8-fold higher (p < 0.05) in patients of the first and second groups as compared to the control group (*Table*). ALT and AST activities also increased in both groups on average 2.5-and 2.9- fold (p < 0.05), respectively. Standard treatment resulted in a 2.1-fold decrease in the γ-GTP activity (p < 0.05), and 1.5- and 1.4-fold decrease in the ALT and AST activities, respectively. The activity of hepatocyte damage marker enzymes changed more significantly in the second group of patients receiving the combination therapy including melaxen. Thus, the γ-GTP activity decreased 2.8-fold (p < 0.05), ALT activity decreased 1.8-fold (p < 0.05), and AST activity decreased 1.6-fold (p < 0.05).

**Table:** Effects of basic therapy and combination therapy including melaxen on liver function parameters in patients with the acute stage of chronic alcoholic hepatitis

Group		Liver function parameters		
		γ-GTP, µkat/L	ALT, nmol/(s∙L)	AST, nmol/(s∙L)
Control group, normal values (n = 65)		0.88 ± 0.04	95.9 ± 13.7	52.5 ± 7.3
Group 1, basic therapy (n = 28)	Before treatment	3.34 ± 0.14*	241.6 ± 19.3*	151.6 ± 10.8*
	After treatment	1.63 ± 0.06**	161.8 ± 16.2**	108.9 ± 11.1**
Group 2, combination therapy including melaxen (n = 24)	Before treatment	3.33 ± 0.12*	256.1 ± 14.6*	152.2 ± 10.4*
	After treatment	1.19 ± 0.04**	145.1 ± 11.3**	96.3 ± 13.7**

*Note*. The difference between the parameter and its reference value (*) or the value in the group of patients after treatment (**) is statistically significant at p < 0.05. Reference values of enzyme activity in males: γ-GTP – (0.25–1.77) μkat/L; ALT – normal (28–189) nmol/(s∙L); AST – (28–127) nmol/(s∙L).

**Fig. 1:**
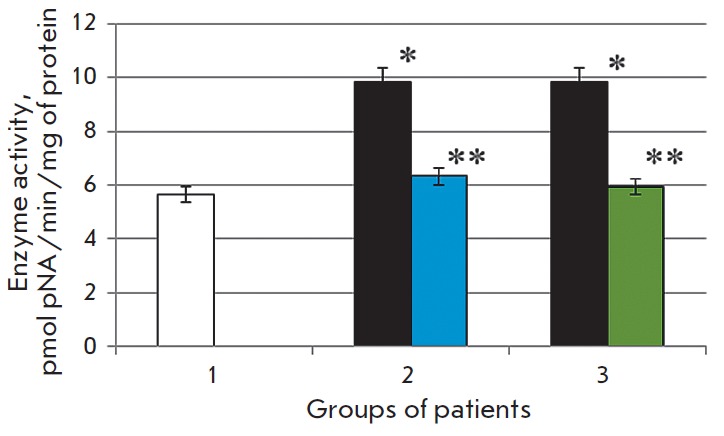
Activity of caspase-1 in the blood serum in the normal state (1) in patients with chronic alcoholic hepatitis after standard therapy (2), in the case of the combination therapy including melaxen (3): before (blue) and after treatment (green). Note: The accuracy of the values (p) ≤ 0.05 (*) – compared with the normal value (**) – compared with the pathology.

**Fig. 2:**
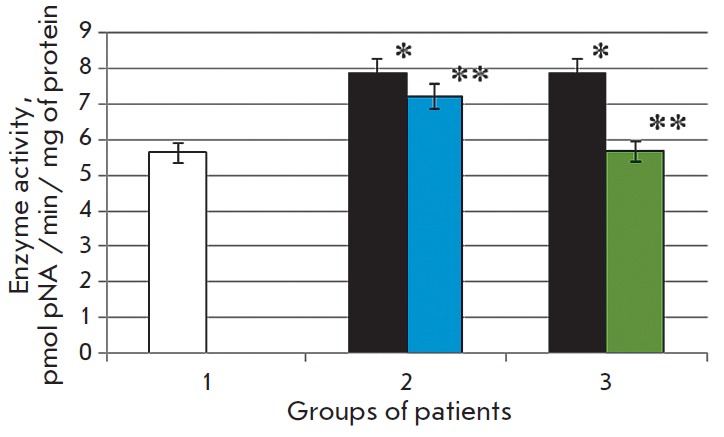
Activity of caspase-3 in the blood serum in the normal state (1) in patients with chronic alcoholic hepatitis after standard therapy (2), in the case of the combination therapy including melaxen (3): before (blue) and after treatment (green). Note: The accuracy of the values (p) ≤ 0.05 (*) – compared with the normal value (**) – compared with the pathology.

The study revealed that the development of CAH in patients was associated with 1.7- and 1.4-fold increases in the caspase-1 and caspase-3 activities (p < 0.05), respectively (*Fig. 1, Fig. 2*), which is indicative of intensification of apoptotic processes. Basic therapy resulted in changes in the caspase activity toward normal values. Thus, caspase-1 activity decreased 1.6-fold, and caspase- 3 activity decreased 1.1-fold (p < 0.05) compared to the results obtained before treatment (*Fig. 1, Fig. 2*). A more pronounced decrease in the activities of both caspase-1 (1.7-fold) and caspase-3 (1.4-fold) (p < 0.05) (*Fig. 1, Fig. 2*) was observed in the group of patients who received melaxen along with the conventional treatment, which apparently was associated with the correction of the melatonin level under the action of this drug.

**Fig. 3:**
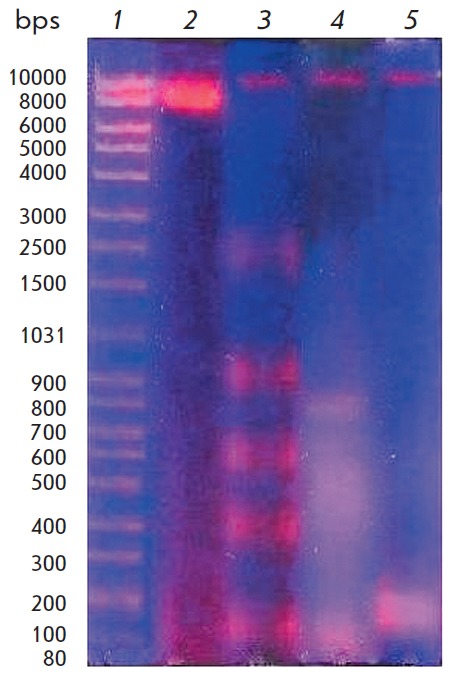
Electrophoregram of DNA from the blood leukocytes of the patients: control group (2), patients with CAH before treatment (3), after the conventional therapy (4), and after combined therapy including melaxen. Lane 1 shows DNA marker

The data on the changes in the caspase activity in CAH patients are consistent with the results of the assessment of the fragmentation degree of blood leukocyte DNA in patients. According to the results of a electrophoretic analysis, DNA was represented by a single fragment at the beginning of the track (*Fig. 3*) in the blood samples from the control group donors. DNA isolated from the leukocytes of CAH patients was fragmented compared to DNA from the control samples. The degree of DNA fragmentation decreased after the standard treatment. DNA fragmentation was barely visualized in most blood samples from patients receiving melaxen along with the basic therapy.

**Fig. 4:**
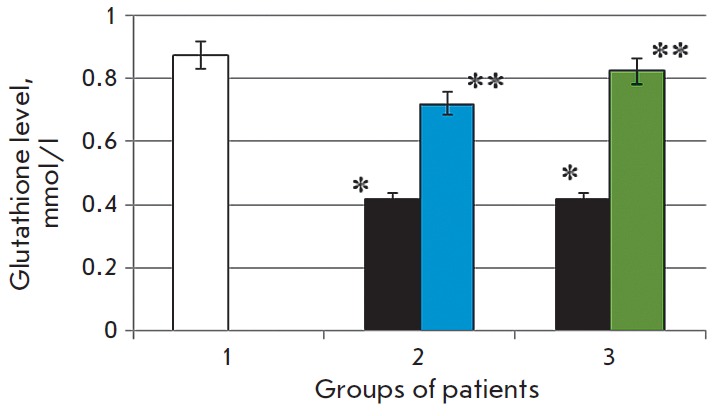
The reduced glutathione level in the blood serum of the control group patients (1), patients with chronic alcoholic hepatitis after standard therapy (2), in the case of combination therapy including melaxen (3): before treatment (blue), after treatment (green). Note. The accuracy of the values (p) ≤ 0.05 (*) – compared with the normal value (**) – compared with the pathology.

The serum GSH level in the first group of CAH patients decreased on average 2.1-fold (p < 0.05) compared to the control level (*Fig. 4*) before the administration of hepatoprotectors. It is known that alcohol induces oxidative stress and damages the liver cells [22]. Obviously, activation of free-radical oxidation associated with this process reduces the GSH level. We observed a 1.7-fold increase in GSH concentration (p < 0.05) after basic treatment as compared to the values obtained before treatment.

The GSH level was 2.1 times lower (p < 0.05) in the second group of patients than that in the control group. Concentration of this metabolite increased after the combination therapy including melaxen and became equal to that of the control group (2.1-fold) (*Fig. 4*).

**Fig. 5:**
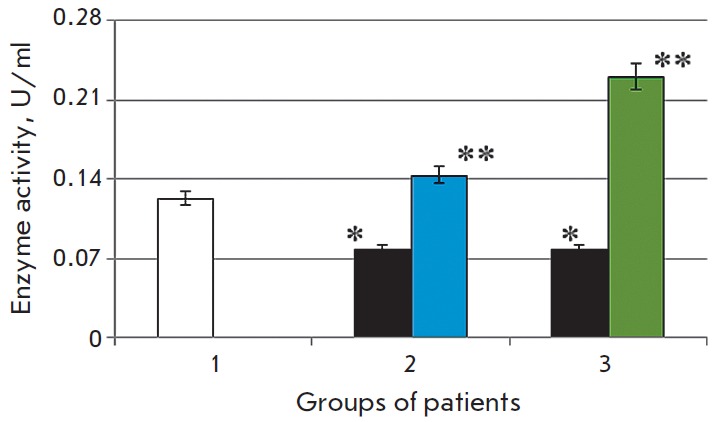
Glutathione peroxidase activity in terms of E per ml (A) and specific enzyme activity (B) in patients with chronic alcoholic hepatitis after the standard therapy (2), in the case of the combination therapy including melaxen (3) before treatment (blue), after treatment (green). Note. The accuracy of the values (p) ≤ 0.05 (*) – compared with the normal value (**) – compared with the pathology.

**Fig. 6:**
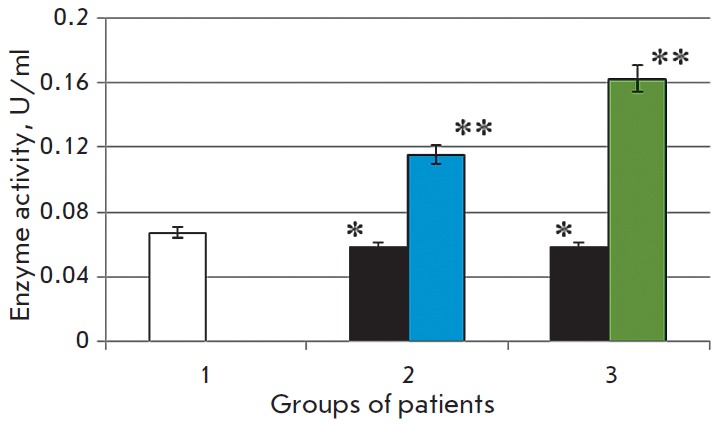
Glutathione reductase activity in terms of E per ml (A), and specific enzyme activity (B) in patients with chronic alcoholic hepatitis after the standard therapy (2), in the case of the combination therapy including melaxen (3) before treatment (blue), after treatment (green). Note. The accuracy of the values (p) ≤ 0.05 (*) – compared with the normal value (**) – compared with the pathology.

Our study revealed that the GP and GR activities in the serum of CAH patients of the first group decreased on average 1.6-fold (p < 0.05) and 1.2-fold (p < 0.05), respectively, before the administration of the basic treatment as compared to the control level (*Fig. 5, Fig. 6*). The decrease in GR activity in CAH patients apparently can contribute to the decrease in the GSH level. After the standard treatment, the GP and GR activities increased on average 1.8-fold (p < 0.05) and 2.0-fold (p < 0.05), respectively, as compared to the values prior to the basic therapy.

In the second group of CAH patients, the GP and GR activities decreased prior to the therapy within the same range as in the first group. The GP and GR activities increased 2.9- and 2.8-fold, respectively, after combination therapy including melaxen. Thus, the most significant increase in GP/GR- system activity was observed in this group of patients (*Fig. 5, Fig. 6*).

The GST activity decreased 1.6-fold (p < 0.05) in the first group of CAH patients prior to the administration of hepatoprotectors as compared to the control level. Obviously, the decrease in the GST activity was caused by a significant consumption of reduced glutathione in response to excessive formation of ROS due to oxidative stress induced by CAH. This hypothesis is consistent with the observed increase in the GST activity along with the increase in the GSH level after treatment. Thus, the enzyme activity increased 1.5-fold (p < 0.05) after the basic therapy including the administration of hepatoprotectors.

**Fig. 7:**
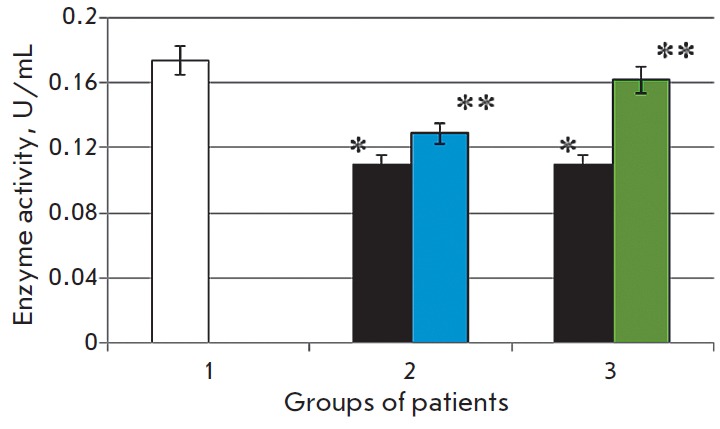
Glutathione transferase activity in terms of E per ml (A), and specific enzyme activity (B) in patients with chronic alcoholic hepatitis after the standard therapy (2), in the case of the combination therapy including melaxen (3) before treatment (blue), after treatment (green). Note. The accuracy of the values (p) ≤ 0.05 (*) – compared with the normal value (**) – compared with the pathology.

In the second group of CAH patients, the GST activity prior to the therapy varied within the same range as in the first group. The GST activity increased 1.8-fold (p < 0.05) after the combination treatment including the administration of hepatoprotectors and melaxen as compared to the results before the treatment. Thus, the administration of melaxen resulted in a more significant increase in the GST activity as compared to the first group of patients (*Fig. 7*).

**Fig. 8:**
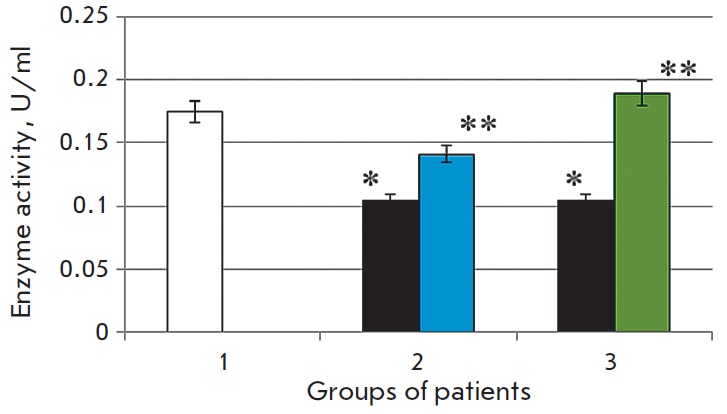
NADP-isocitrate dehydrogenase activity in terms of E per ml (A) and specific enzyme activity (B) in patients with chronic alcoholic hepatitis after the standard therapy (2), in the case of the combination therapy including melaxen (3) before treatment (blue), after treatment (green). Note. The accuracy of the values (p) ≤ 0.05 (*) – compared with the normal value (**) – compared with the pathology.

Changes in the activities of NADPH-generating enzymes in CAH patients and after treatment were revealed. It was found that the serum activity of NADPIDG decreased on average 1.7-fold in the groups of CAH patients as compared to the control group. NADP-IDG activity increased on average 1.4-fold after the basic treatment as compared to the values before treatment. In the case of combination therapy including melaxen, enzymatic activity increased more significantly and was 1.8 times higher than the activity before treatment (*Fig. 8*).

**Fig. 9:**
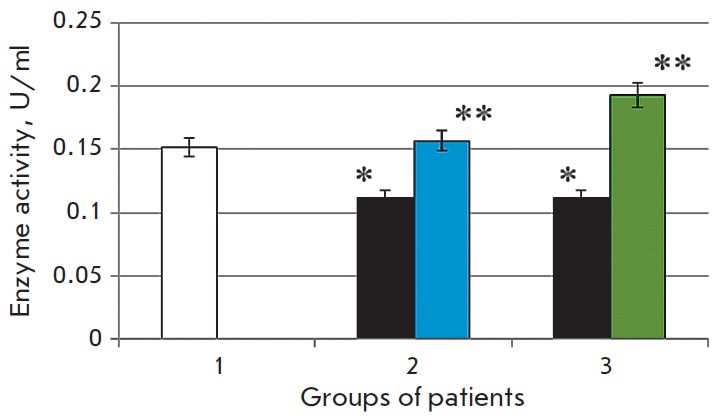
The activity of glucose-6-phosphate dehydrogenase in terms of E per ml (A) and specific enzyme activity (B) in patients with chronic alcoholic hepatitis after the standard therapy (2), in the case of the combination therapy including melaxen (3) before treatment (blue), after treatment (green). Note. The accuracy of the values (p) ≤ 0.05 (*) – compared with the normal value (**) – compared with the pathology.

G6PDG activity decreased on average 1.4-fold (p < 0.05) in CAH patients. The activity increased 1.4- fold after the standard therapy as compared to the results before treatment (*Fig. 9*). The combination therapy including melaxen on average led to a 1.7-fold increase in G6PDG activity in the second group of patients with the acute stage of CAH (*Fig. 9*).

The decrease in the activities of NADPH-generating enzymes apparently could be one of the reasons for the decrease in the GR activity in CAH patients.

It should be noted that in several studies on animal models, the activity levels of antioxidant enzymes, including glutathione AOS, NADPN-generating enzymes, as well as the parameters showing the intensity of free-radical processes (biochemiluminescence parameters, DC level), correlated with those in the liver, and with the status of liver damage, as assessed by the activity of marker enzymes (ALT, AST) [23-27].

## DISCUSSION

Biochemical parameters of liver functions (*Table*) confirm that CAH is associated with the metabolic disorders in hepatocytes and their damage is accompanied by cell cytolysis and the release of ALT, AST and γ-GTP in blood. The decrease in the values of the investigated parameters confirms the hepatoprotective effect of the basic treatment. More pronounced changes in parameters in the second group of patients suggests that inclusion of melaxen to the basic therapy, which corrects melatonin levels in the body, enhanced the hepatoprotective effect apparently due to the antioxidant and immunostimulatory effect of this hormone.

The increase in caspase activity in the serum of CAH patients (*Fig. 1 and Fig. 2*) was apparently associated with excessive generation of ROS in this pathology. Thus, the development of hepatocyte apoptosis was observed in the experimental models of alcohol-induced liver diseases [28]. Furthermore, the experimental hepatitis induced by concanavalin A was associated with the increased activity of caspase-3 that was in particular due to liver-infiltrating lymphocytes, which are subjected to activation-induced apoptosis [29]. The reduced activity of both caspases after the basic therapy was apparently due to the fact that current treatment reduced the rate of ROS generation and inhibition of apoptotic processes. More significant changes in the activities of caspase-1 and caspase-3 in patients who received the combination therapy including melaxen were probably due to the correction in the melatonin level during the administration of this drug. It is known that melatonin reduces oxidative damage to lipids, DNA and mitochondria [30], and it increases the expression of antiapoptotic genes that belong to the Bcl-2 group, protecting lipids from peroxidation and cells from subsequent apoptosis [31].

The results in determining DNA fragmentation in the blood leukocytes of CAH patients are consistent with the data on change in the caspase activity in CAH, during the standard treatment, and melaxen intake along with basic therapy. DNA extracted from the blood samples of CAH patients was significantly fragmented. According to some researchers, such fragments are produced due to the action of apoptosis-specific nucleases in the terminal phase of apoptosis [32]. DNA degradation at first produces large fragments of approximately 300 kbps, and later – 30–50 kbps. The next step produces fragments of 180 bps or their multiples by the internucleosomal degradation of the DNA due to the action of CAD (caspase-activated DNase) calcium-sensitive endonuclease. It is these fragments that are detected by electrophoresis as the “apoptotic ladder.” It is known that such DNA fragmentation can be related to proteolytic cleavage by caspases and DNA topoisomerase II, which participates in DNA supercoiling. Furthermore, H1 histone, which protects DNA from endonuclease action at the internucleosomal level, is the substrate of caspases during apoptosis [33]. The electrophoretic analysis of DNA extracted from the blood of CAH patients revealed a band at the molecular- weight range corresponding to degraded DNA that is characteristic of necrosis [34]. A decrease in DNA fragmentation was observed after the conventional therapy, indicating a positive effect of the treatment. Inclusion of melaxen in the basic therapy significantly reduced the degree of DNA fragmentation, which may be indicative of an anti-apoptotic action of this drug.

A decrease in the serum GSH level in CAH patients of more than 2-fold with respect to the control (*Fig. 4*) is apparently due to the imbalance between the rate of free-radical processes and the antioxidant system activity. It is known that reduced glutathione plays a key role in the low-molecular-weight thiol antioxidant system. It effectively inactivates ROS and is the most sensitive component in the overall scheme of nonspecific resistance of the body under oxidative stress conditions [35]. In addition, glutathione is involved in the conversion of antioxidants, such as ascorbic acid, α-tocopherol, thioctic acid, ubiquinone, in maintaining the optimal structural and functional state of biological membranes and regulating the synthesis of heat shock proteins [36]. As noted above, the microsomal enzymes of the monooxygenase system, in particular SUR 2E1, not only oxidize alcohol, but also can transform xenobiotics into toxic metabolites. Concomitant activation of free-radical oxidation apparently leads to the depletion of the GSH level, which demonstrates that the ability of the liver to neutralize toxic compounds is impaired.

After the basic treatment, the GSH concentration was 1.7 times higher than that before treatment, which is apparently due to the decrease in the intensity of freeradical oxidation (FRO), and, as a result, a decrease in the consumption of this metabolite owing to the positive treatment effect. The combination treatment including melaxen facilitated the recovery of the glutathione concentration to its normal level, which is obviously associated with the powerful antioxidant effect of melatonin and its positive effect on the glutathione system (*Fig. 4*).

Research into the functioning of glutathione antioxidant system enzymes has shown that chronic alcohol intoxication is associated with a decrease in the serum activity of selenium GP and GR, which is also indicative of the decreased antioxidant status of patients. The decrease in the GP activity is probably due to the decrease in selenium concentration in chronic alcohol intoxication. It is known that the activity of selenium GP depends on the selenium level. An insufficient selenium level results in the inhibition of enzyme activity. A reduced activity of the enzyme associated with a deficiency of selenium is due to the decrease in the amount of GP mRN A [37]. Selenium is required for the synthesis of selenocysteine, which is part of the active center of the enzyme and plays an important role in catalysis [38]. GP functioning is closely related to the functioning of GR. Since the reaction catalyzed by GR yields a quickly mobilizable source of GSH, it is likely that a decrease in the GR activity can significantly contribute to the decrease in the concentration of this thiol in CAH patients.

The increased activity of the GP/GR-system after the standard treatment of CAH patients was apparently associated with the positive effect of the basic therapy on the antioxidant status of the patients. GP and GR activities increased even more significantly after the combination therapy including melaxen (*Fig. 5, Fig. 6*). The antioxidant activity of melatonin apparently can be associated with the activation of antioxidant enzymes and/or stimulation of their synthesis [39].

The significant decrease in GST activity that was revealed in the serum of CAH patients compared to the normal activity could obviously be associated with a significant consumption of the reduced glutathione in response to an excessive formation of ROS during the development of the oxidative stress induced by the pathological condition. It is known that GST uses the reduced glutathione for conjugation with hydrophobic substances, their reduction or isomerization. GSH is an essential component in the reaction of neutralization of the toxic products of lipid peroxidation, reduction of lipid hydroperoxides, and the biotransformation of xenobiotics catalyzed by multifunctional GST [14]. In this regard, the decreased GSH levels in CAH could well lead to a reduced activity of GST. This hypothesis is consistent with the increasing GST activity, along with the increasing GSH level after treatment. The increase in GST activity was likely associated with the positive effect of the treatment on the antioxidant status of patients, including the decrease in GSH consumption. Notably, the administration of melaxen facilitated a more significant increase in the GST activity as compared to the first group of patients (*Fig. 7*).

It was found that CAH is also associated with an altered activity of NADPH-generating enzymes (NADPIDG and G6PDG). NADP-IDG activity decreased to a greater extent than G6PDG activity. A less significant decrease in G6PDG activity is probably attributable to the role of the pentose phosphate pathway as a supplier of reducing equivalents for fatty acid biosynthesis, which is activated in the liver cells under conditions of its fatty degeneration in chronic alcohol intoxication. It is known that it is G6PDG, being the key enzyme of the pentose phosphate pathway, that is responsible for the bulk of the NADPN required for the synthesis of fatty acids [40]. The decrease in the activity of NADPN-generating enzymes could be due to the negative effect of ROS, which is excessively generated in the pathological state. There is evidence of inhibition of the activity of some glycolysis enzymes in patients with chronic alcohol intoxication, which is accompanied by an increase in the hepatic glucose and lactate levels [41]. The decrease in the activity of the enzymes involved in the transformation of tricarboxylic acids, and NADP-IDG in particular, can apparently occur under these conditions (*Fig. 8*).

A more significant increase in the activity of NADPIDG and G6PDG in the blood of patients who received drug capable of melatonin level correction as compared to those receiving the standard treatment could be due to the induction of enzyme synthesis under the action of this hormone. It is known that melatonin can increase the expression of some of the enzymes involved in the antioxidant defense of the body [42].

It should be noted that the decrease in the activity of NADPN-generating enzymes could be one of the reasons for the decrease in the GR activity in CAH patients. Moreover, a significant increase in GR activity after the basic treatment and an even more significant recovery of enzyme activity during the administration of melaxen were associated with an increase in the G6PDG and NADP-IDG activities under these conditions. The increased reference level of the activity of the GR and NADPN-generating enzymes that was observed under these conditions could be due to the strong effect of antioxidant therapy, which facilitates AOS mobilization under oxidative stress conditions and becomes systemic in chronic alcohol intoxication. It is likely that melatonin (its level is corrected under the action of melaxen) can act as an adaptogen regulating the activity of the glutathione system, as well as enzymes capable of generating NADPN, in accordance with the exposure of the body to disease-producing factors.

## CONCLUSIONS

Inclusion of melaxen to the CAH therapy enhances the hepatoprotective and membrane-stabilizing effects. This fact has been confirmed by the parameters that characterize the functioning of the liver; in particular, aminotransferases and γ-GTP. This is apparently due to the antioxidant properties of melatonin, which is included as a component of melaxen. A combination therapy using melaxen led to a more significant decrease in the development of apoptotic processes in CAH patients, as evidenced by a more significant decrease in the activity of caspase-1 and caspase-3, and the DNA fragmentation degree, than that in patients who received the conventional therapy. Correction of the melatonin level in the body leads to significant recovery in the GSH level, activity of glutathione group enzymes of AOS (GR, GP, GST, as well as G6PDG and NADP-IDG NADPN-generating enzymes) as compared to such parameters during the basic treatment. The findings suggest the effective protective action of melaxen in toxic liver injury, which has a favorable impact on the state of free-radical homeostasis and significantly reduces the severity of cytolytic hepatocyte injury.

## Abbreviations

LPO: lipid peroxidation
ROS: reactive oxygen species
GSH: reduced glutathione
GR/GP system: glutathione reductase / glutathione peroxidase system
CAH: chronic alcoholic hepatitis
FRO: free-radical oxidation
GP: glutathione peroxidase
GR: glutathione reductase
GST: glutathione-S-transferase
G6PD: glucose-6-phosphate dehydrogenase
AOS: antioxidant system
NADP-IDH: NADPisocitrate dehydrogenase
